# Attitudes towards a multimodal precision medicine algorithm for predicting treatment response in depression: findings from a large cross-sectional European survey

**DOI:** 10.3389/fpsyt.2025.1642511

**Published:** 2025-11-04

**Authors:** Viktor T. H. Wahner, Johannes C. S. Zang, Rosa Glaser, Britta Kelch, Inga Stonner, Martina Contu, Mara Dierssen, Ewa Ferensztajn-Rochowiak, Massimo Gennarelli, Dobrochna Kopeć, Mirko Manchia, María Martínez de Lagrán, Valentina Menesello, Oumayma Meskini, Alessandra Minelli, Pasquale Paribello, Júlia Perera Bel, Giulia Perusi, Marco Pinna, Claudia Pisanu, Marie-Claude Potier, Filip Rybakowski, Ferran Sanz, Alessio Squassina, Silke Jörgens, Bernhard T. Baune

**Affiliations:** ^1^ Department of Psychiatry, University of Münster, Münster, Germany; ^2^ Department of General Internal Medicine and Psychosomatic, University Hospital Heidelberg, Heidelberg, Germany; ^3^ Section of Psychiatry, Department of Medical Sciences and Public Health, University of Cagliari, Cagliari, Italy; ^4^ Centre for Genomic Regulation (CRG), The Barcelona Institute of Science and Technology, Barcelona, Spain; ^5^ Department of Adult Psychiatry, Poznan University of Medical Sciences, Poznan, Poland; ^6^ Department of Molecular and Translational Medicine, University of Brescia, Brescia, Italy; ^7^ Genetics Unit, San Giovanni di Dio Fatebenefratelli Center (IRCCS), Brescia, Italy; ^8^ Department of Pharmacology, Dalhousie University, Halifax, NS, Canada; ^9^ Paris Brain Institute (ICM), National Centre for Scientific Research (CNRS), Paris, France; ^10^ Research Programme on Biomedical Informatics (GRIB), Hospital del Mar Research Institute (IMIM), Barcelona, Spain; ^11^ Department of Mental Health and Addiction Services, ASST Spedali Civili of Brescia, Brescia, Italy; ^12^ Section of Neuroscience and Clinical Pharmacology, Department of Biomedical Sciences, University of Cagliari, Cagliari, Italy; ^13^ Department of Medicine and Life Sciences, Universitat Pompeu Fabra, Barcelona, Spain; ^14^ Department of Psychiatry, Dalhousie University, Halifax, NS, Canada; ^15^ Department Hamm 2, Hochschule Hamm-Lippstadt, Hamm, Germany; ^16^ Florey Institute of Neuroscience and Mental Health, Parkville, VIC, Australia; ^17^ Department of Psychiatry, University of Melbourne, Parkville, VIC, Australia

**Keywords:** major depressive disorder (MDD), treatment resistant depression (TRD), precision medicine, personalized medicine, precision psychiatry, treatment response, algorithm

## Abstract

**Background:**

Precision medicine aims to facilitate a more individualized treatment selection and a more accurate diagnosis. While there is broad ranging research on precision psychiatry and the corresponding computational tools, its concepts and implementation are underway, little is known about the attitudes towards the actual use of precision psychiatry tools in the management of major psychiatric disorders, such as Major Depressive Disorder (MDD). This study aims to investigate the attitudes of depressive patients, professionals (physicians, psychologists and scientists) and the general population towards a novel, multimodal precision medicine algorithm designed to predict antidepressant treatment response.

**Methods:**

5490 participants from 21 European countries, consisting of three groups of stakeholders, patients with depression (n= 421), professionals (n = 367) and the general population (n = 4702), were polled with a newly developed cross-sectional survey. A hypothetical decision scenario was used to examine the participants’ attitudes, in which they were asked for their approval or disapproval for the application of a multimodal precision medicine algorithm to predict treatment response in antidepressant-treatment.3

**Results:**

The general population had an acceptance rate of 78.8%. Overall, 74.6% of patients with MDD would agree to undergo testing using the multimodal algorithm in their current situation and 80.2% reported they would have done so at the time of their first diagnosis. In contrast, the psychiatrist’s acceptance rates towards a multimodal algorithm were higher when patients had been in treatment for some time (79.3%) compared to those who had only recently been diagnosed (55.2%). This pattern was present across all other specialties within the professionals group. A considerable number of participants wished to receive more information before deciding, but few declined its application altogether. All groups indicated an openness towards personalized treatment options in general.

**Conclusion:**

Overall, participants indicated a large degree of acceptance towards the application of a multimodal precision medicine algorithm. Although limited by the hypothetical nature of the decision scenario, this study provides valuable perspectives from different stakeholders. Future research should move beyond attitudes and address further implementation hurdles that need to be overcome for the successful implementation of novel precision psychiatry approaches in psychiatric care.

## 1 Introduction

Major Depressive Disorder (MDD) is a highly prevalent psychiatric condition, affecting approximately 4.4% of the world population (1). It is considered one of the leading causes of disability worldwide, placing a fundamental burden on healthcare systems (2). Symptoms of MDD vary between patients, they include depressed mood, anhedonia, fatigue, sleep disturbances, changes in appetite, cognitive impairment and, in severe cases, life-weary thoughts. Patients are usually treated with pharmacotherapy in the form of antidepressants. Despite a large selection of antidepressants, many patients do not benefit sufficiently from pharmacotherapy. Indeed, finding the right antidepressant and dosage often follows a trial-and-error approach, causing delayed symptom relief. An estimated proportion of up to 30% of patients do not sufficiently respond to antidepressant-treatment, a condition often referred to as Treatment Resistant Depression (TRD). While the exact definition is a matter of debate (3, 4), TRD is often clinically defined as the failure to achieve remission after the treatment with at least two adequately dosed antidepressants. TRD is acknowledged as a severe mental illness, with high and often unmet treatment needs, placing a high economic burden on healthcare systems (5).

In response to these challenges, precision medicine, and precision psychiatry particularly has gained attention as an innovative approach to optimize psychiatric pharmacotherapy prescription (6–8). Precision medicine intents to approach patients’ needs on a customized level by taking individual genetic profiles, life-style variables, environmental factors and clinical courses into account. A compelling application of precision psychiatry is the prediction of treatment response (9). This approach seems particularly promising in the context of TRD, where the need to develop more personalized therapy options has been emphasized (4): non-responding patients, who are susceptible to later develop TRD, could be identified at an early stage, enabling early treatment adaptations and even prevention (10, 11). The development of a multimodal precision medicine algorithm combining clinical, omics and sex-related data in order to predict treatment response in MDD is the objective of the EraPerMed-scheme funded project “Toward PrecisiOn Medicine for the Prediction of Treatment response in major depressive disorder through stratification of combined clinical and -omics signatures” (PROMPT) (10), which this work is part of. Furthermore, PROMPTs objective is to evaluate the degree of acceptance of the multimodal algorithm and its potential application among patients with depression, general population and various stakeholders involved in MDD management (10). This effort is key for its possible implementation into clinical practice.

Indeed, research investigating attitudes towards the application of precision medicine algorithms in psychiatry in general, and in MDD management in particular, is scarce and an open-minded attitude towards its use has largely been assumed within the field. Previous surveys studying the degree of acceptance of personalized treatment approaches in psychiatry as well as in other specialties focused on pharmacogenetic (PGx) testing. A large body of literature suggest a high degree of acceptance of PGx-testing among the public (12–14) and patients (15–17), while professionals range from more moderate to very optimistic attitudes (18–23). Using PGx-testing to guide antidepressant prescription has been key in personalized depression treatment and its clinical utility has been demonstrated (24), for example testing individuals for metabolizing enzymes, aiming to minimize drug interactions and adverse effects and to improve drug response. However, the next generation of precision psychiatry evolves beyond single PGx-applications to multimodal algorithms incorporating clinical, lifestyle, psychosocial variables, and omics, aiming to provide even more precise assistance in personalized drug prescription. It is of great interest to study whether these novel precision medicine tools are similarly accepted as the PGx-tests. In order to address this development, a large-scale survey exploring the perspective by various stakeholders, including patients, professionals and the broader public, is necessary. Investigating the general opinion is valuable, as these insights are key for the public debate, policymaking, educational purposes as well as the research community, seeking ways to facilitate the implementation into real-world clinical settings.

Concluding, the aim of this publication is, first, to provide an overview of the data structure of the surveys conducted in PROMPT and, second, to assess the acceptance of a novel multimodal algorithm designed to predict antidepressant treatment response at the descriptive level, using a hypothetical decision scenario, targeting three groups of stakeholders: patients, professionals and the general population.

## 2 Methodology

### 2.1 Survey development and distribution

As part of the PROMPT consortium a new cross-sectional survey was developed to target three groups: the general population, patients and professionals (including psychiatrists, psychologists, other medical specialties and scientists) (10).

The survey used a hypothetical decision scenario to assess the attitudes towards a novel predictive tool for treatment response. It further included questions regarding shared decision making, gene-environment interaction, genetic knowledge, expertise and personal experience with MDD and body-mind-dualism. The objective of this work was to analyze the stakeholders’ attitudes towards the multimodal algorithm. Therefore, only items within the scope of this study are described in detail in the following section. To adequately address each target group, survey items were adjusted accordingly. Some questions were modified or added to address the participants’ respective background, thus resulting in three different survey versions: one survey addressing the general population, one survey addressing the patients and one survey addressing professionals. Additionally, the professionals’ survey was adapted for specific subgroups of professions (psychiatrists, psychologists, scientists, other medical specialties). A pilot survey was distributed to evaluate its rationale and length. The final survey versions consisted of single-choice, multiple-choice and Likert-scale questions with an approximate total duration of 45 minutes.

To reach a wide European audience the surveys were translated from English into German, Polish, Italian, French, and Spanish using DeepL. The translations were then reviewed and refined by native speakers from the respective participating PROMPT Study group site based on the English version. The survey was implemented and distributed using REDCap, a secure web-based platform for electronic data capture (25). In addition, a paper-pencil version was used to address patients in the clinical context. The entire data collection took place between January 2023 and January 2025. Participants had to be at least 18 years old to be eligible for participation.

The distribution of each survey version was customized to maximize reach within each target group: The survey link for the general population survey was disseminated using newsletters, social media platforms, online platforms and print media, including newspaper articles and posters. Professionals were contacted via institutional and clinic newsletters, flyers at conferences and outpatient professionals were also directly addressed via email. The survey targeting patients was conducted at three PROMPT consortium sites (Münster, Germany; Poznan, Poland; Cagliari, Italy).

### 2.2 Survey measures

For all three target groups demographic information (age, gender, country of residence, religiosity) was collected at the beginning of the survey. The general population was asked for their personal experiences with depression, while the patients provided information detailed in their medical history. Professionals were asked about professional experiences with depression.

Next, participants’ attitudes towards the multimodal algorithm were evaluated. To introduce the topic, subjects were given a brief background information:

‘Treatment with antidepressant medication (antidepressants) is often an important part of depression treatment and can help to significantly improve symptoms. Yet antidepressants do not work for 3–5 out of 10 people. This is only noticed after having tried different medications and combinations of medications over a longer period, leading to a treatment failure with potential unnecessary side effects.’

Following this, the novel, multimodal precision medicine tool was explained:

‘A newly developed test can predict whether antidepressants will work for a person. The test uses information from DNA and RNA extracted from blood cells. This information is then combined with clinical and personal information, such as age and sex. The test result indicates if a patient with depression likely benefits from antidepressant medication or not before starting any medication.’

Note that the term ‘test’ (in the sense of a standard medical test procedure) was used instead of ‘algorithm’ to ensure a simpler, more accessible language for the subjects. Finally, the question towards the hypothetical scenario of being tested with the multimodal algorithm was framed as:

‘Imagine you have been diagnosed with depression. Would you like your doctor to test you with the procedure described above to find out whether antidepressants are a suitable treatment option for you?’

Possible single-choice answers were: *‘Yes, I would like my doctor to test me’*, *‘No, I don’t want my doctor to test me’* or *‘I need more information to decide this.’* Subsequently, subjects rated how easy it was to make this decision were (*‘This decision was easy for me’*) and whether they considered it meaningful (*‘This is a significant decision’*). Both items were answered on a 7-Point Likert scale ranging from 1 (*‘I completely disagree’*) to 7 (*‘I completely agree’*).

The hypothetical decision scenario described above was applied to the population survey and was adapted for the other target groups. The patient survey avoided the hypothetical phrasing and instead asked the patients two questions, first about their current situation (‘*In your current situation, would you like your doctor to test you using the procedure described above to see if antidepressants are an appropriate treatment option for you?’*) and second, from a retrospective perspective *(‘Please put yourself back in the situation when you were first diagnosed with depression and first steps of treatment were discussed. Looking back, would you recommend your younger self to take the test?’).*

The professionals’ survey included questions from the treatment perspective, also considering two temporal viewpoints: ‘*Would you suggest the test to a patient of yours that only recently has been diagnosed with depression?’* and ‘*Would you suggest the test to a patient of yours that has been in treatment for depression for some time?’*. Physicians (psychiatrists, neurologists, etc.) were asked if they would actively suggest the test, while psychologists were asked whether their patient would benefit from it. The scientist group received the same scenario as the general population. Response options remained the same across surveys. The professionals had no follow-up questions regarding significance and ease of the decision.

At last, all participants across all surveys were asked about their general treatment preferences, indicating their agreement on a Likert-scale ranging from 1 (*‘I completely disagree’*) to 7 (*‘I completely agree’*) to the following two statements: First, *‘If I had a disease, I would choose the therapy that has been used so far for most people with the same disease.’* and second, *‘If I had a disease, I would choose the therapy that was* sp*ecifically designed just for me.’*.

### 2.3 Data analysis

All statistical analyses were conducted using JASP (Version 0.19.3) (26). Descriptive statistics, including means (M), standard deviations (SD), maximum (Max), minimum (Min) values and frequencies (acceptance rates), were computed for demographic variables and survey responses. For all Likert-scaled questions medians (Mdn) were additionally computed, to better capture central tendency. The analysis was performed for each survey type. The professionals’ acceptance rates were analyzed by background: psychiatrists, psychologists, other specialties and scientists. The differences between the backgrounds (for example, psychologists would not be authorized to assign the test) were considered in the survey and did not allow an overarching analysis as one group regarding the decision scenario. Additionally, the acceptance rates in the decision scenario were calculated for each country. Due to low participation, country differences were not calculated across the distinct professional backgrounds within the professionals group. Of all participants that started the survey only those who at least answered the items related to the decision scenario were included into data analysis. Participants from outside of Europe were excluded from the analysis.

## 3 Results

### 3.1 Sample characteristics

In total, 6020 participants started the online survey, with n = 5241 completing a sufficient number of questions to be included into the analysis, resulting in an overall dropout rate of 13% (n = 779). Additionally, we received 329 paper-pencil surveys, resulting in an overall sample size of n = 5570. A total of 80 surveys were answered from countries outside of Europe and were excluded in this analysis. Thus, resulting in the final sample size of N = 5490.

Overall, the survey received answers from 21 different countries within Europe. Most subjects n = 5416 or 98.7% of the sample were from Germany (n = 2294), Italy (n = 1841), Poland (n = 526), Spain (n = 563), and France (n = 192). An additional 74 participants from other European countries, combined in the analysis as ‘others’, were from Austria (n = 14), Belgium (n = 5), Denmark (n = 1), Estonia (n = 1), Greece (n = 1), Hungary (n = 2), Latvia (n = 1), Malta (n = 1), The Netherlands (n = 9), Norway (n = 3), Portugal (n = 2), Serbia (n = 4), Slovakia (n = 1), Sweden (n = 2), Switzerland (n = 12), and The United Kingdom (n = 14).

The largest group polled was the general population with a final sample size of n = 4702. It consisted of 75% female and 21% male subjects, and the mean age was 38.9 years (SD = 16.8), ranging from 18 to 93 years. 35% of the general population sample stated that they were diagnosed with MDD by a professional, 37% had a relative and/or a friend that suffered from MDD. Only 11.6% stated to have no personal experience with depression at all. A summary of the sociodemographic characteristics is presented in **Table 1**.

**Table 1:** Demographic characteristics of the general population (n = 4702).

Age	M = 38.87 (SD = 16.84) (Min = 18; Max = 93)
Gender	
Female	3530 (75.1)
Male	1111 (20.6)
Other	42 (0.9)
Not stated	19 (0.4)
Religious	
Yes	1447 (30.8)
No	2808 (59.7)
Not stated	447 (9.5)
Experience with depression	
Diagnosed by professional	1651 (35.1)
Friend and/or Relative diagnosed	1742 (37)
No experience	511 (11.6)
Country of residence	
Germany	1777
Italy	1784
Poland	401
Spain	553
France	127
Other	60

Absolute numbers, percentages in brackets.

In the MDD patient sample (n = 421), eleven patients had to be excluded during the analysis because they did not answer the decision-scenario item (seven from Germany and four from Poland), leaving a final sample of n = 410. The sample consisted of 57% women and 41% men. The mean age of the patients was 41.95 years (SD = 15.20), with a range from 18 to 75 years. The sample presented the following clinical characteristics: the mean age at first diagnosis of MDD was 32.54 years (SD = 14.25; Min = 11; Max = 73) and 89.5% reported an antidepressant intake. The majority had been undergoing treatment for several years with 26.1% having been treated for over nine years. 20.2% reported having tried either no or at most one antidepressant. The rest tried at least two drugs, with 28% having tried over five antidepressants. Finally, the sample indicated to be burdened by MDD, answering the question *‘Overall, how much do you feel burdened by your depression?’* with a mean of 5.60 (SD = 1.45; Mdn = 6) on a 7-point Likert scale (1= *‘not burdened at all’*; 7 = *‘very strongly burdened’*). **Table 2** summarizes the demographics and clinical details of the patient sample.

**Table 2:** Demographic and clinical characteristics of the patient group (Maximum n = 410).

Demographic characteristics	
Age	M = 41.95 (SD = 15.20) (Min = 18; Max = 75)
Gender	
Female	234 (57)
Male	168 (41)
Other	4 (1)
Not stated	4 (1)
Religious	
Yes	117 (28.5)
No	241 (58.8)
Not stated	52 (12.7)
Country of residence	
Germany	320 (78)
Italy	30 (7.3)
Poland	60 (14.6)
Clinical characteristics	
Age – first episode experienced	M = 27.72 (SD = 15.80) (Min = 6; Max = 73)
Age – first Diagnosed	M = 32.54 (SD = 14.25) (Min = 11; Max = 73)
Family history of depression	
Yes	213 (52)
No	115 (28)
Unknown	80 (19.50)
Not stated	2 (0.50)
Years being in MDD-treatment	
0 to 6 months	79 (19.3)
7 to 12 months	46 (11.2)
1 to 3 years	89 (22.7)
4 to 7 years	64 (15.6)
7 to 9 years	24 (5.9)
> 9 years	107 (26.1)
Current intake of antidepressants	
Yes	367 (89.5)
No	41 (10)
Unknown	2 (0.5)
Total number of antidepressants tried (Lifetime)	
0	17 (4.1)
1	66 (16.1)
2 to 3	126 (30.7)
4 to 5	82 (20)
> 5	115 (28)
No stated	4 (1)
Experienced Burden through MDD*	5.60 (1.45) Mdn = 6
Suicide attempted	
Yes	94 (22.9)
No	287(70)
Not stated	29 (7.1)

*Absolute numbers, percentages in brackets.* *7-point Likert scale Item (1 = not burdened at all; 7 = very burdened)

The professionals’ sample (n = 367) consisted of 116 psychiatrists, 122 psychologists and 97 scientists. Further six neurologists and 26 general practitioners summarized as ‘other specialty’ (n = 32) answered the survey. Overall, the professionals’ mean age was 44.46 (SD = 12.58) years, ranging from 21 to 78 years. 63.2% percent of the professionals were female. The highest female proportion was in the psychologist-group with 81.1%, which also had the lowest mean age of all professional subgroups (M = 41.55; SD = 11.85; Min = 24; Max = 73). 48.3% of the psychiatrists answering the survey were female and 50.9% were male. Their mean age was 46.09 years (SD = 12.48; Min = 25; Max = 75). Most psychiatrists (94.8%), psychologists (90.2%) and physicians from the other specialties (93.8%) were currently treating patients suffering from MDD. For details of the professional’s cohort with its respective subgroups see **Table 3**.

**Table 3:** Professionals survey sample – characteristics for each background.

Variable	Professionals (Overall) (n = 367)	Psychiatrists (n = 116)	Psychologists (n = 122)	Other Speciality* (n = 32)	Scientists (n = 97)
Age	M = 44.46 (SD = 12.58) (Min = 21; Max = 78)	M = 46.09 (SD = 12.48) (Min = 25; Max = 75)	M = 41.55 (SD = 11.85) (Min = 24; Max = 73)	M = 49.06 (SD = 11.41) (Min = 21; Max = 71)	M = 44.66 (SD = 13.18) (Min = 21; Max = 78)
Gender					
Female	232 (63.2)	56 (48.3)	99 (81.1)	15 (46.9)	62 (63.9)
Male	132 (36)	59 (50.9)	22 (18)	17 (53.1)	34 (35.1)
Other	–	–	–	–	–
Not stated	3 (0.8)	1 (0.9)	1 (0.8)	–	1 (1)
Religious					
Yes	108 (29.4)	33 (28.4)	35 (28.7)	15 (46.9)	25 (25.8)
No	217 (59.1)	64 (55.2)	77 (63.1)	14 (43.8)	62 (63.9)
Not stated	42 (11.4)	19 (16.4)	10 (8.2)	3 (9.4)	10 (10.3)
Treating MDD patients		110 (94.8)	120 (90.2)	30 (93.8)	–
Country of residence					
Germany	190	63	74	28	25
Italy	27	17	9	–	1
Poland	61	24	33	3	1
Spain	10	4	1	1	4
France	65	2	1	–	62
Other	14	6	4	–	4

Absolute numbers, percentages in brackets. *Consisting of Neurologists (n = 6) and General Practitioners (n = 26).

### 3.2 Decision scenario: acceptance of the multimodal precision medicine algorithm

**Table 4** summarizes the acceptance rates of all groups. In the general population sample 78.8% indicated willingness to undergo testing, 17.6% stated that they required more information before making the decision and 3.6% decided against taking the test. The decision in this hypothetical scenario was considered meaningful (M = 5.98; SD = 1.53; Mdn = 7) and easy to make (M = 5.84; SD = 1.62; Mdn = 7) as more than half of respondents scored a 7 on the 7-point Likert-scale. The acceptance rate varied across countries but was not lower than 70% in any group (**Table 5**). The polish sample, which was also the youngest of all countries analyzed (M = 25.22; SD = 8.80), had the highest acceptance rate (88.3%). The largest groups polled, Italy and Germany, had acceptance rates of 80.6% and 75.4%, respectively. Additionally, the attitudes toward the test were calculated for participants of the general population that reported a diagnosis of MDD and those without reporting such diagnosis. As depicted in **Table 4**, the group with reported MDD showed a higher willingness to take the test (81.2%) compared to the group not indicated to be diagnosed with depression (77.5%).

**Table 4:** Overview of all acceptance rates by group.

Group	Yes	No	More information
General population (n = 4702)			
Overall	3705 (78.8)	169 (3.6)	828 (17.6)
Diagnosed Depression (n = 1651)	1340 (81.2)	57 (3.5)	254 (15.4)
Without MDD (n =3051)	2365 (77.5)	112 (3.7)	574 (18.8)
Patients (n = 410)			
Current Episode	306 (74.6)	29 (7.1)	75 (18.3)
When first diagnosed	328 (80.2)	20 (4.9)	61 (14.9)
Professionals (n = 367)			
Psychiatrists (n = 116)			
Recently diagnosed	64 (55.2)	12 (10.3)	40 (34.5)
In treatment for some time	92 (79.3)	6 (5.2)	18 (15.5)
Psychologists (n = 122)			
Recently diagnosed	50 (41.0)	8 (6.5)	64 (52.5)
In treatment for some time	71 (58.2)	2 (1.6)	49 (40.2)
Other Specialities (n = 32)			
Recently diagnosed	15 (46.9)	5 (15.6)	12 (37.5)
In treatment for some time	18 (56.3)	4 (12.5)	10 (31.3)
Scientists (n = 97)	80 (82.5)	1 (1.0)	16 (16.5)

Absolute numbers, percentages in brackets.

**Table 5:** Overview of acceptance rates by countries.

Country	n	Age	Gender				Test acceptance			Test acceptance (Retrospective)		
			f	m	o	n.s.	Yes	No	M.I.	Yes	No	M.I.
General population												
Germany	1777	38.35 (16.29)	1358 (76.4)	394 (11.2)	18 (1)	7 (0.4)	1339 (75.4)	77 (4.3)	361 (20.3)	–	–	–
Italy	1784	38.88 (16.24)	1312 (73.5)	450 (25.2)	13 (0.7)	9 (0.5)	1437 (80.6)	60 (3.4)	287 (16)	–	–	–
Poland	401	25.22 (8.80)	315 (78.6)	78 (19.5)	6 (1.5)	2 (0.5)	354 (88.3)	6 (1.5)	41 (10.2)	–	–	–
Spain	553	53.33 (15.08)	424 (76.7)	129 (23.3)	–	–	443 (80.1)	14 (2.5)	96 (17.4)	–	–	–
France	127	28.57 (11.84)	82 (64.6)	41 (32.3)	3 (2.4)	1 (0.8)	90 (70.9)	7 (5.5)	30 (23.6)	–	–	–
Other	60	33.55 (13.94)	42 (70)	5 (8.3)	13 (21.7)	–	42 (70)	5 (8.3)	13 (21.7)	–	–	–
Patients												
Germany	320	41.08 (15.58)	168 (52.5)	146 (45.6)	4 (1.3)	2 (0.6)	234 (73.1)	25 (7.8)	61 (19.1)	254 (79.6)	19 (6)	46 (14.4)
Italy	30	51.63 (12.50)	22 (73.3)	8 (26.7)	–	–	20 (66.7)	2 (6.7)	8 (26.7)	22 (73.3)	0 (0)	8 (26.7)
Poland	60	41.68 (12.71)	44 (73.3)	14 (23.3)	–	2 (3.3)	52 (86.7)	2 (3.3)	6 (10.0)	52 (86.7)	1 (1.7)	7 (11.7)

Absolute numbers, percentages in brackets. n.s., not stated; M.I., More Information. Patients were asked if they would take the test in their current situation (= Test Acceptance) and

when first diagnosed with MDD (Retrospective).

Next, responses from the patient survey were analyzed regarding their willingness to take the test for both temporal viewpoints (in their current situation and retrospectively, when first diagnosed). The large majority would take the test in their current situation (74.6%) with an additional proportion stating they would have done so retrospectively (80.2%; **Table 4**). In their current situation 18.3% stated the need for more information to decide, only 7.1% rejected to currently undergo testing. For country specific acceptance rates of the patients group see **Table 5**. As the general population, patients rated the decision as both important (M = 5.97; SD = 1.37; Mdn = 6) and easy (M = 5.53; SD = 1.71; Mdn = 6).

Because of differences in the hypothetical decision scenarios, professionals’ attitudes were analyzed for each background separately (**Table 4**). 55.2% of the psychiatrists stated to test recently diagnosed patients and a higher proportion of 79.3% indicated to test patients that had been in treatment for some time. This trend of a higher degree of acceptance of the multimodal algorithm for patients with a longer medical history was also observed in the psychologist-group (recently diagnosed: 41% vs. diagnosed for some time: 58.2%) as well as in the other specialties-group (recently diagnosed: 46.9% vs. diagnosed for some time: 56.3%). Notably, the psychologists had the highest demand for more information, with 52.5% requesting more details before making the decision. The scientists, who were asked the same scenario as the general population, had a high acceptance rate: 82.5% stated they would like to be tested, 16.5% wanted more information and 1% denied taking the test altogether.

### 3.3 Preference for personalized treatment options

**Figure 1:**
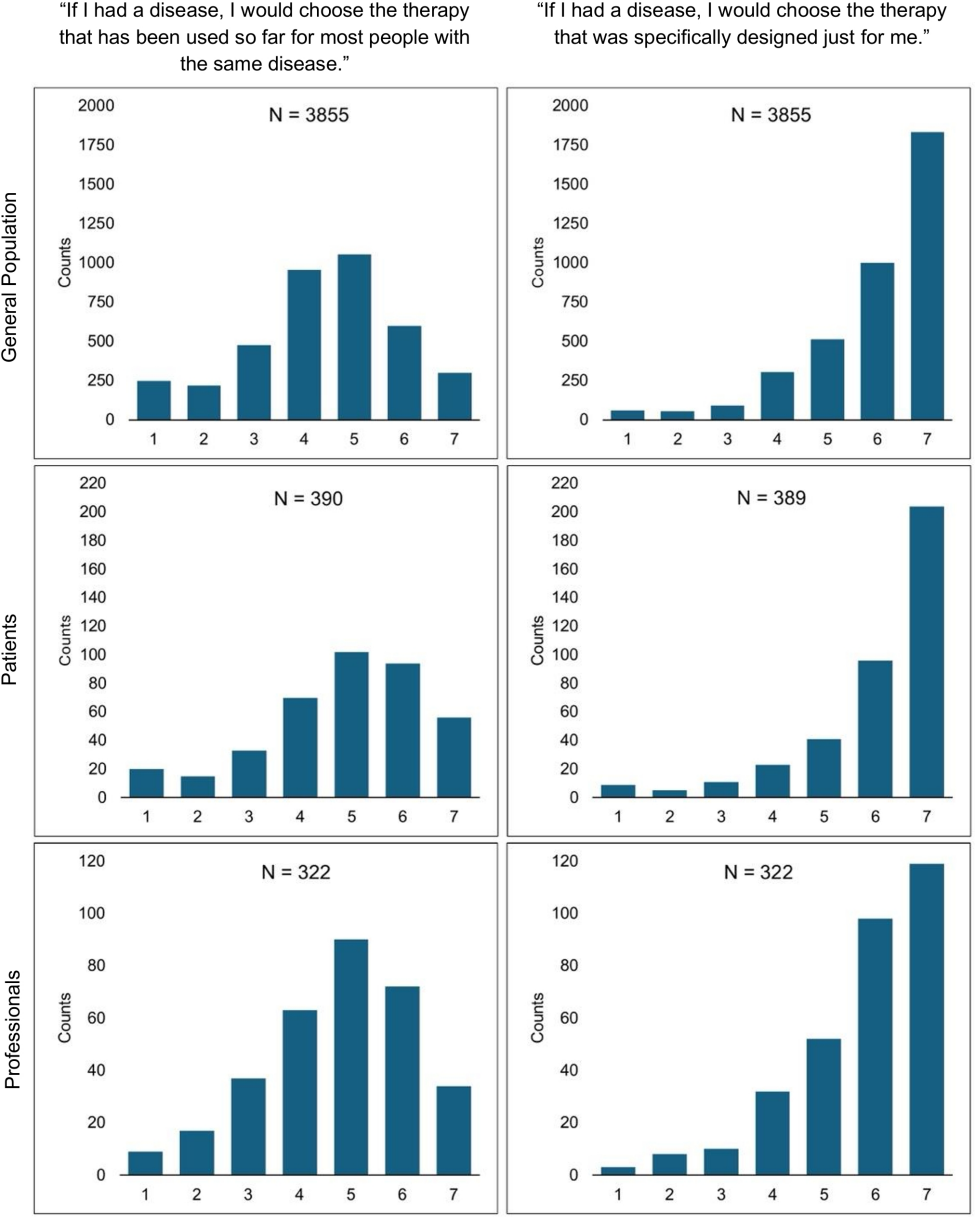
Participants’ agreement towards therapy options. Likert-scale item ranging from 1 = I completely disagree to 7 = I completely agree. Includes only the proportion of participants who answered this item, which explains the differences in sample sizes reported in **Tables 1**–**5**.

The final items assessing preferences for therapy options had a lower response rate with 83.2% (n = 4567) of the included sample answering this part of the survey. All groups indicated higher agreement on the item asking for personalized treatment (General Population: M = 5.98, SD = 1.32; Mdn = 6; Patients: M = 6.05, SD = 1.38; Mdn = 7; Professionals: M = 5.77, SD = 1.33; Mdn = 6) compared to the item asking for the one-fits-all approach (General Population: M = 4.38, SD = 1.51; Mdn = 5; Patients: M = 4.86, SD = 1.58; Mdn = 5; Professionals: M = 4.74, SD = 1.47, Mdn = 5). The distribution of responses on the 7-point Likert scale for each group is displayed in **Figure 1**.

## 4 Discussion

This study presents the initial findings from the large-scale European PROMPT survey, assessing the attitude of the general population, professionals and patients affected from depression towards a novel, multimodal precision medicine algorithm designed for the prediction of antidepressant response. The results indicate a positive opinion towards the multimodal algorithm across all examined groups. To the best of the authors’ knowledge this is the first large-scale survey to poll various stakeholders’ attitudes towards a multimodal precision medicine algorithm in depression treatment combined in one survey.

The results are in line with previous research, examining attitudes towards precision medicine in the context of PGx-testing. Similar to results from other developed countries, such as Belgium (12), Korea (14), and the USA (13), the general population showed an overall positive attitude towards the test, with the vast majority choosing to undergo testing in the decision scenario. This was also evident across all countries independently analyzed, though, acceptance rates varied by country and should be investigated in further analyses. Additionally, participants from the general population that stated to be diagnosed with MDD had an even higher acceptance rate than those with no diagnosis. This indicates that consent could depend on the degree of personal exposure to MDD. Similarly, prior knowledge and health literacy are decision factors that have been mentioned elsewhere (12) and should be examined more thoroughly.

Next, the high acceptance rates found in the patients group was also consistent with previous findings from surveys on the use of PGx (15, 16). Noteworthy, this study adds a new valuable perspective: patients were asked if they would do the test in their current situation as well as retrospectively, when they first received their diagnosis. Interestingly, the acceptance rate was higher in the retrospective view, meaning more patients would have chosen the test when first diagnosed. Though a recall bias cannot be ruled out, from the patient’s perspective, testing prior to the first prescription is seen as especially useful. Patients’ interest in testing might depend on their clinical history. Possibly, patients with longer treatment and history of non-response to antidepressant treatment might be less interested in testing, as they would not benefit from it. Further analyses will be necessary to investigate this hypothesis in detail.

In total, only a small subset of patients expressed unwillingness to undergo testing using the multimodal algorithm. Nonetheless, it is important to address this subgroup to understand the reasons behind this reluctance. Previous studies in the context of PGx in mental health have identified concerns such as results interpretation, financial liability, privacy/data security and stigmatization (27). Similar concerns are also present in the application of precision medicine tools in other medical specialties, emphasizing their broader relevance (28). Follow-up analyses should address these concerns of the patients. In addition, expanding to qualitative investigations is important to gain a holistic understanding of patients concerns, as well as their perceived benefits of the multimodal algorithm.

In comparison to the other stakeholder groups, the professionals who treat MDD patients had more moderate acceptance rates. Interestingly, a higher acceptance rate for running the test with patients that have been treated for some time versus recently diagnosed patients was observed among all groups (psychiatrists, psychologists, other specialties). This result was especially apparent in the psychiatrist-group where the largest difference was observed. Similarly, data from a French cohort of psychiatrists showed a higher interest in running PGx-tests in TRD patients than in MDD patients (20). It appears that clinicians would rather use the test to validate the patients’ clinical course than to test their treatment response before first antidepressant prescription. This confirmatory use of the test however fails its aspired utilization as an assisting tool for clinical decisions, aiming to preemptively test for non-responding patients at an early stage. This result reflects more reserved opinions towards using precision medicine tools at a preemptive stage (29, 30). Addressing this debate is out of scope of this discussion but it is important to mention that future lines of research, ideally including clinical trials, need to investigate the utility and effectiveness of precision medicine algorithms.

It is noteworthy that only a few clinicians declined using the test altogether but rather demanded more information than given in the decision scenario. This appears plausible, assuming that many practitioners may not be familiar enough with the topic to confidently decide for or against the test. Previously, studies revealed a rather low level of expertise among psychiatrists (20, 31). This was also observed among the next generation of clinicians, who stated high interest in precision medicine but felt unsatisfactory education in medical school for its practical application (32). Indeed, precision medicine is usually not part of standard medical or psychological training. The lack of knowledge may also explain the particularly high demand for more information in the psychologists’ group. It is likely that the psychologists have the least expertise in genetics and pharmaceutics in comparison to the other specialties in the professionals group. In summary, training clinicians and students appears to be an important aspect of translating precision medicine tools to clinical practice. Future efforts are needed for the development of dedicated educational strategies, such as offering post-graduate training modules and multidisciplinary workshops in clinical practice or adopting them into standard medical school curricula.

At last, there are several limitations with potential impact on the reported results. This study achieved to poll a considerable number of participants, nevertheless it is not a representative sample. Thus, a self-selection bias must be considered, limiting the external validity of the results to the sample presented here. Next, the translation methods applied in this work did not include back translation procedures, small deviations between language versions may have occurred, which may hinder comparability between countries. A further limiting aspect is the selection of demographic variables. Though not further interpreted in this work, religiosity was collected across all stakeholder groups, and its specific role, especially in combination with beliefs about genetics and mental health should be examined in follow-up analyses. However, previous work pointed out the importance to integrate underrepresented populations in precision medicine research (33, 34). Administering additional demographic variables like marital status, parenthood or ethnicity is important to gain a more profound knowledge about the sample, enabling better understanding of societal nuances within the population. This, of course, is also of great significance in the development of predictive precision psychiatry tools (11). Furthermore, an important limitation to this study is the use of a hypothetical decision scenario. The scenario does not include information on validity and reliability of the test but rather presupposes its functionality and predictive power, possibly explaining to some extend the high acceptability among respondents. This is particularly important in the general population, who may not have had prior exposure to this topic. This limitation seems to be supported by the result that in all groups the majority found the decision very easy to make. It appears natural to decide with ease for a beneficial procedure if it is free of uncertainties. The predictive abilities of the test have previously been reported as an important decision factor for or against taking a genetic test in the context of mood disorders (35) and need to be communicated with patients, as false hopes and misunderstandings are a potential harm for this group. Finally, it should be acknowledged that this study was intentionally limited to a descriptive and thus exploratory rationale, providing a foundation for subsequent detailed analyses, with inferential conclusions being beyond its scope.

There is a significant gap between the development of precision medicine tools in the field of psychiatry and their implementation in real-world clinical settings (36). Structural, financial and regulatory barriers delay the transition from development to broad clinical implementation. Moreover, the development of precision medicine algorithms still need to navigate methodological challenges (37). Data protection and ethical considerations relating to the use of sensitive genetic and personal health data present further hurdles (38, 39).

Nevertheless, the results of this study imply an overall desire for more personalized therapy options, as across all groups the approval for personalized therapies was higher than for conventional ones. However, the successful implementation will require overcoming barriers beyond attitudes, including cost, data security and regulatory approval. Taking together, the findings of this study suggest that precision psychiatry holds immense potential and is met with open attitudes among various stakeholders, at the same time its successful implementation will also depend on overcoming substantial systemic challenges.

## 5 Conclusion

The survey results point towards expectations that precision medicine tools in psychiatry may soon improve clinical decision making, identifying most suitable treatments at an early stage and ultimately enhancing psychiatric care. A successful translation to the real world is only possible by carefully considering the perspectives of various stakeholders. By offering new insights, this work contributes to this endeavor. Future lines of research should include clinical trials, to examine the use of the multimodal precision medicine algorithm and the stakeholders’ attitudes under real world conditions. In addition, qualitative approaches should be employed to obtain more detailed insights into the concerns and refusal attitudes of those affected. Finally, health-economic evaluations are needed in future studies, adding another valuable stakeholder perspective and to better capture healthcare complexity. All in all, this study shows a large degree of acceptance of a multimodal precision psychiatry algorithm for antidepressant response among the public, patients, psychiatrists and other professionals. Further this work facilitates in-depth follow-up analyses of the here introduced PROMPT survey, which are necessary for a better understanding of factors influencing the decision making in the stakeholder groups and countries examined.

## Data Availability

The raw data supporting the conclusions of this article will be made available by the authors, without undue reservation.
